# Behaviour of freshwater snails (*Radix balthica*) exposed to the pharmaceutical sertraline under simulated predation risk

**DOI:** 10.1007/s10646-017-1880-6

**Published:** 2018-01-18

**Authors:** Melanie Lea Hedgespeth, Tomasz Karasek, Johan Ahlgren, Olof Berglund, Christer Brönmark

**Affiliations:** 1 Aquatic Ecology, Department of Biology, Lund University, Ecology Building, Lund, 223 62 Sweden; 2 Department of Hydrobiology, Faculty of Biology, Biological and Chemical Research Centre, University of Warsaw, Żwirki i Wigury 101, Warsaw, 02-089 Poland

**Keywords:** Pharmaceuticals, Behavioural toxicology, Freshwater toxicology, Snail, Predation, Boldness

## Abstract

Due to their potential for affecting the modulation of behaviour, effects of selective serotonin reuptake inhibitors (SSRIs) in the environment are particularly interesting regarding interspecies interactions and non-consumptive effects (NCEs) induced by predator cues in prey organisms. We evaluated the effects of sertraline (0.4, 40 ng/L, 40 µg/L) over 8 days on activity and habitat choice in the freshwater snail *Radix balthica*, on snails’ boldness in response to mechanical stimulation (simulating predator attack), and their activity/habitat choice in response to chemical cues from predatory fish. We hypothesised that sertraline exposure would detrimentally impact NCEs elicited by predator cues, increasing predation risk. Although there were no effects of sertraline on NCEs, there were observed effects of chemical cue from predatory fish on snail behaviour independent of sertraline exposure. Snails reduced their activity in which the percentage of active snails decreased by almost 50% after exposure to fish cue. Additionally, snails changed their habitat use by moving away from open (exposed) areas. The general lack of effects of sertraline on snails’ activity and other behaviours in this study is interesting considering that other SSRIs have been shown to induce changes in gastropod behaviour. This raises questions on the modes of action of various SSRIs in gastropods, as well as the potential for a trophic “mismatch” of effects between fish predators and snail prey in aquatic systems.

## Introduction

Pharmaceutical residues are increasingly detected in aquatic environments, especially in surface water samples from areas that serve as recipients of wastewater treatment discharges (e.g. Kolpin et al. 2004). Active ingredients of antidepressant pharmaceuticals such as the selective serotonin reuptake inhibitors (SSRIs) have been detected in wastewater recipients within the nanogram- to low microgram-per-liter range (e.g. fluoxetine: 20 ng/L, citalopram: 40 ng/L, venlafaxine: 1000 ng/L, sertraline: 49 ng/L; Schultz and Furlong 2008), and furthermore, these compounds have been shown to affect behaviour in aquatic organisms, mainly fish (reviewed by Brodin et al. 2014). However, aquatic invertebrates including crustaceans and the less-frequently studied molluscs have recently been identified as being particularly sensitive to SSRIs due to low effect concentrations and high bioaccumulation potential (Fong and Ford 2014; Meredith-Williams et al. 2012).

The neurotransmitter serotonin has been identified as a modulator of biological activity in both gastropods and crustaceans. Influence on locomotion (Pavlova 2001; Syed and Winlow 1989), reproductive processes (Diefenbach et al. 1998; Manger et al. 1996), and predator vigilance behaviour (Il-Han et al. 2010) has been documented in gastropods, and in crustaceans, effects on reproductive processes (Wongprasert et al. 2006), locomotion (Tain et al. 2006), and social behaviour (Huber and Delago 1998) have been reported. Though the exact mechanisms are still unknown, it is thought that SSRIs are linked to serotonin activity in these invertebrate groups (e.g. Campos et al. 2013). For instance, SSRIs have been shown to affect both reproduction and behaviour in crustaceans (Campos et al. 2012; Guler and Ford 2010), and reproduction in gastropods (Couper and Leise 1996; Nentwig 2007). However to date, comparatively little research on the potential effects of SSRIs on behaviour or locomotion in gastropods have been reported (but see Fong et al. 2015). Because data are particularly scarce regarding aquatic invertebrate exposure to the SSRI sertraline (as opposed to other SSRIs such as fluoxetine), we selected sertraline as the compound of interest for this study.

In recent years, numerous studies have indicated that predator-induced changes in behaviour can have considerable effects in food chains and that these non-consumptive effects (NCEs) can be as strong as or even stronger than the direct, lethal effects of predators (e.g. Bernot and Turner 2001; Werner and Peacor 2003). Thus, there is a need for increased understanding of how freshwater organisms respond behaviourally to the presence of environmental contaminants such as antidepressants in order to be able to predict their ecological consequences in recipient ecosystems. Previous research has indicated that the serotonergic system mediates changes in snail behaviour caused by predators, examined via injection of serotonin antagonists into organisms (Il-Han et al. 2010). Due to their potential for affecting the modulation of behaviour, effects of SSRIs that are found in aquatic systems are particularly interesting with regard to intra- and interspecies interactions and NCEs. Predator–prey interactions would be a suitable interaction to consider in this regard, since pollutant-induced behavioural changes that may seem subtle or go unnoticed in single-species systems may nevertheless have profound effects on population and community dynamics in the environment.

To advance environmental risk assessment, ecotoxicity testing has been shifting from the explicit use of acute, high-dose exposures for assessing mortality to the inclusion of chronic exposures assessing effects of contaminants on ecologically-relevant life history parameters, and ultimately, population growth rates (Forbes and Calow 2002). However, the use of behavioural endpoints is still uncommon with regard to the ecotoxicity testing of various pharmaceuticals, and endpoints that reveal information on organism interactions all the more so. In particular, studies on aquatic systems have focused on behavioural endpoints regarding organism interactions within exposed vertebrate taxa (e.g. Barry 2014; Brodin et al. 2013; Hedgespeth et al. 2014; Weinberger and Klaper 2014), but so far, relatively few have addressed invertebrates in this sense (but see Brodin et al. 2014; Fong and Ford 2014).

In this study, we evaluated the effects of the SSRI sertraline on the behavioural changes of the freshwater snail *Radix balthica* in response to chemical cues from predatory fish. These snails occur in a range of habitats: from small, ephemeral ponds and streams to large lakes and rivers; subsequently, they are also exposed to a variety of different predators (e.g. Brönmark 1992; Nyström et al. 1999). In order to reduce vulnerability they have evolved a number of adaptive traits against predation including changes in life history strategies (Brönmark et al. 2012; Crowl and Covich 1990), morphology (DeWitt et al. 2000; Hoverman et al. 2005), and behaviour (Brönmark and Malmqvist 1986; Rundle and Brönmark 2001; Turner 1996). One effective response of freshwater snails to chemical cues emitted by predators is to increase their refuge use, i.e. via hiding under stones or crawling up above the water’s surface (Rundle and Brönmark 2001; Turner 1996). Such predator-induced changes in habitat use and activity have further been found to have strong effects on the biomass of periphytic algae, the snails’ primary food source (Brönmark 1989), indicating the importance of such NCEs for ecosystem-wide processes.

In recent years it has become increasingly evident that not all individuals respond similarly to an environmental cue; there is large variability among individuals within a species and even within a population (Bolnick et al. 2011). Many studies have demonstrated that individuals show consistent differences in behavioural traits, including aggression (Duckworth 2006), risk-taking (van Oers et al. 2005), activity (Kurvers et al. 2009), and exploration (Minderman et al. 2009). Such individual-level differences in behaviour that are consistent over time and context, known as “animal personalities,” have been demonstrated across a wide range of taxa, from mammals to insects (Bell et al. 2009). One of the most commonly-studied aspects of personality is the behavioural variation of organisms along a bold-shy continuum, in which bolder animals have a higher propensity to engage in risky behaviours and shy individuals are consistently more risk-averse and cautious (Wilson et al. 1994). Recent studies have shown that *R. balthica* demonstrate consistent individual variation in boldness (Ahlgren et al. 2015). Hence, we suspect that the potential effects of SSRIs on such behaviours in snails (in terms of altering the mean response or the consistency of the behaviours within individuals) could consequently impact predator–prey interactions, i.e. via the NCEs induced by predators.

We have therefore assessed the effect of sertraline and predator cue exposure on the activity, boldness, and avoidance behaviour of the freshwater snail *Radix balthica*. Snails were exposed to sertraline (0.4, 40 ng/L, or 40 µg/L) over 8 days and then subjected to either a simulated, mechanical predator cue or an chemical predator cue from molluscivorous fish (*Carassius carassius*). Effects on behaviours were assessed as a result of the potential interaction between the two types of stressors, and as a result of sertraline exposure or predator cue alone. Overall, we expected that sertraline exposure would impact the snails’ behaviours in response to predator cues in a negative manner, i.e. in a manner that would make them more susceptible to predation in natural systems.

## Methods

### Study organisms

Adult freshwater snails *Radix balthica* (Lymnaeidae; 1.7 ± 0.55 cm length) were collected from ponds located in areas that are not directly impacted by wastewater effluent in the vicinity of Lund, southern Sweden. They were allowed to acclimate to 16:8 h light:dark, 18 °C conditions (similar to environmental conditions), held in dechlorinated tap water, and fed ad libitum with rabbit chow three times per week for 14 days prior to the experiment. Two molluscivorous crucian carp (*Carassius carassius*, ~10 cm body length) were collected by trap-netting in a pond in the University Park, Lund and held in an aerated 100 L aquarium under the same conditions as the snails for approximately 2 months prior to the experiment. The carp were fed snails (*R. balthica* and *Lymnaea stagnalis*) three times per week during holding, occasionally supplemented with rabbit chow, and fresh, dechlorinated tap water was added weekly (approximately ¼ change; water was not changed directly prior to the experiment). They were fed ten crushed *R. balthica* ~4 h prior to collection of water from the aquarium for use as predator cue in trials on snail behaviour, as predators that have fed on conspecifics elicit stronger behavioural responses in prey compared to unfed predators (Turner et al. 2006).

### Chemical exposure and setup

Stock solutions of sertraline hydrochloride (CAS #79559-97-0, Toronto Research Chemicals Inc.) dissolved in 100% dimethyl sulfoxide (DMSO) were prepared via dilution series. Though we were unable to directly measure exposure medium at the time of the experiment, analysis of the stock solution confirmed the presence of sertraline—a 10× dilution of the highest stock concentration was analysed (nominal: 2.0 mg/L; measured: 2.2 mg/L) using a Xevo G2 Q-TOF (Waters, Sweden) connected to an Acquity UPLC system (Waters, Sweden) according to the method by Boström et al. (2016). These were spiked into 2 L of dechlorinated tap water (pH: 8.1 ± 0.2) resulting in nominal exposure concentrations of 0.4, 40 ng/L, or 40 µg/L sertraline, along with a solvent control treatment. This resulted in a final solvent concentration of 0.002% v-v DMSO:H_2_O in all exposure treatments, which is both below OECD’s recommendations for maximum solvent concentration and is the volume recommended by Hutchinson et al. (2006).

The experiment was performed as a single, static sertraline exposure in 5 L glass containers with 2 L of exposure medium and 5 containers per treatment concentration. Additionally, a 10 × 10 cm ceramic tile was placed into each container, raised 12 mm from the bottom with rubber legs to provide potential refuge for the snails for behavioural assays (see following section). On Day 0 of the exposure, five snails were randomly added to each container (100 snails total). The total duration of chemical exposure was 8 days, during which snails were fed three times with rabbit chow ad libitum. Water was neither changed nor aerated due to the snails being air-breathing and containers were not cleaned during the exposure to avoid any potential removal of sertraline from the system. Based on data from our lab where no significant abiotic degradation of sertraline in water was recorded over 35 days (Boström et al. 2016), and literature data reporting limited biodegradation even in sewage sludge (Styrishave et al. 2011), sertraline was not expected to undergo significant degradation over the 8-day exposure period in our study.

### Behavioural trials

Prior to Day 0 of the exposure, snails were individually tagged and assayed twice during the acclimation period (i.e. at the ends of the first and second weeks directly prior to the experiment) for preliminary boldness scores, using the method described by Ahlgren and coauthors (2015). In short, individuals were placed into a petri dish containing clean water and after a 15 min acclimation period, they were gently tapped on the shell with a pipet tip. After full retraction into the shell, each snail was timed for the re-emergence of both antennae from the shell, with an upper time limit of 180 s. On Day 7 of exposure, this assay was repeated to assess post-exposure boldness, during which snails were placed in a petri dish containing treatment water from their respective containers and after which snails were placed back into their respective exposure containers.

During Days 1–7 of the exposure, activity and location of individuals within the containers were noted three times daily (~11:00, 13:00, and 15:00). Any dead individuals were immediately removed from containers and this information was recorded. Activity was assigned as binary score based on the movement, or lack thereof, of snails at the time of observation, resulting in a percentage score per container. Location was scored as one of three possibilities—under the ceramic tile in the bottom of the container: “under tile;” climbed to or above the surface of the water: “surface;” or in the open: “open” (this included snails that were freely floating, those crawling on the sides of the container but below the water’s surface, and those on the surface of the ceramic tile).

On Day 8 of the experiment, snails were assayed for activity and location as previously described to serve as a control prior to the addition of predator cue. Immediately afterward, 0.1 L of predator cue (water taken directly from the aquarium containing carp previously fed with snails) was added to each treatment container. After 20 min, snails were again assayed to determine activity and location in the presence of the fish cue (as established in the method by Ahlgren and Brönmark 2012). Because Ahlgren and Brönmark determined that water containing carp cue elicits predator-induced behavioural changes in snails (2012), and fish cue was ‘produced’ in the same way in both experiments, chemistry parameters of water containing cue were not measured.

### Statistical analyses

All statistical analyses were carried out using R version 3.1.0 (2014). Data that did not meet the parametric assumptions of normality and homoscedasticity were transformed if necessary, and ranks were used in cases for which parametric assumptions were not met after transformation. Mauchly’s Test for sphericity was also used for repeated measures analyses. For analyses on data regarding habitat choice (i.e. the three possible locations of snails), *p*-values were adjusted using Bonferroni correction.

For activity and location data (Days 1–7), only the snails in the control treatment were analysed to determine whether the time of day the observations were taken had an effect (one-way ANOVA; factor: time of day, block: day nested within time). There was no statistically significant effect of time of day on activity or locations (Activity 45 ± 32%, *F*_2,16_ = 0.0048; Locations: in open 63 ± 28%, *F*_2,16_ = 0.062; at surface 28 ± 29%, *F*_2,16_ = 0.090; under tile 8.3 ± 13%, *F*_2,16_ = 0.31; adjusted *p* > 0.1 in all cases), so further statistical analyses on these endpoints for Days 1–7 used the 15:00 data only. The percentage of active snails and percentages observed in each location over the 7-day exposure were analysed using a two-way repeated measures ANOVAs (factors: sertraline treatment × day; within subjects error: day nested within container).

For Day 8 behavioural data, we assessed the effects of sertraline treatment without and with predator cue on snail activity and locations using 2 × 2 mixed factorial ANOVAs with repeated measures on cue (factors: sertraline treatment × cue; within subjects factor: cue nested within container). For those analyses in which interaction terms were not significant, the interaction term was removed to examine main effects of sertraline and cue only.

To test for pre-exposure, individual consistency in boldness, we used Spearman’s rank correlation on the re-emergence times from the shell. Post-exposure boldness scores (Day 7) and the relative percent changes in individual boldness scores from Day 0 to Day 7 were both analysed using one-way ANOVAs (factor: sertraline treatment; within subjects factor: individual snails nested within container).

## Results

### Effects of sertraline on *R. balthica* behaviour

Mortality of snails at the end of the experiment was assessed using a GLM (Poisson distribution with log link), indicating no significant effect of sertraline treatment on mortality (24 ± 17% in the control; sertraline coefficient = 0.000013, SE = 0.000010, *z* = 1.3, *p* > 0.1). The mean percentage of active snails ranged between 35 and 47% throughout the exposure period, but there was no interaction between time and treatment using a significance threshold of *α *= 0.05 (*F*_18,96_ = 1.6, *p* = 0.088; Supplementary material Fig. S1). Further, there was no main effect of sertraline on snail activity (*F*_3,16_ = 0.29, *p* > 0.1). Likewise, there was no significant effect on sertraline on snail location during the experiment (Supplementary material Fig. S2; in open *F*_3,16_ = 1.5, at surface *F*_3,16_ = 0.74, under tile *F*_3,16_ = 0.66, adjusted *p* > 0.1 in all cases).

### Effects of sertraline on *R. balthica* behaviour when exposed to predator cue

**Fig. 1:**
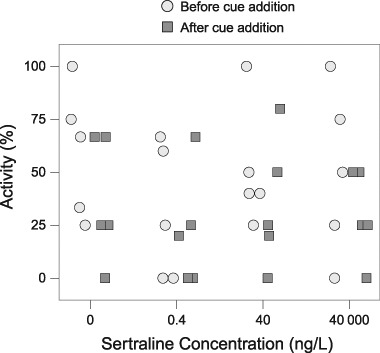
Percentage of active snails in all treatments on Day 8 of sertraline exposure before (light grey circles; overall mean 48%) and after (dark grey squares; overall mean 26%) the addition of chemical cues from the fish predator *Carassius carassius*, with no effect of sertraline treatment (*p* > 0.1). There was a significant effect of cue overall (*p* = 0.038; not indicated in the figure)

**Fig. 2:**
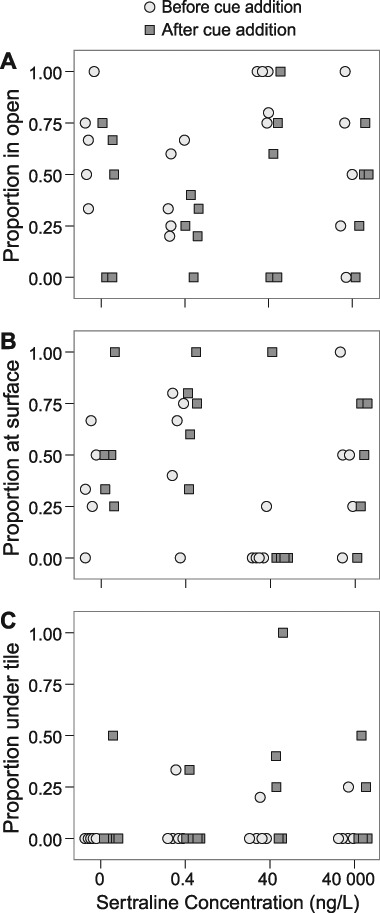
Proportion of snails in all treatments on Day 8 of sertraline exposure before (light grey circles) and after (dark grey squares) the addition of chemical cues from the fish predator *Carassius carassius*, based upon location in containers indicative of habitat choice: **a** in the open (62 and 37% before and after cue, respectively, adjusted *p* = 0.011); **b** at/above the surface of the water (34% before and 47% after cue addition, adjusted *p* > 0.1); and **c** underneath the tile (4% before and 16% after cue addition, adjusted *p* = 0.096). There were no significant effects of sertraline nor interaction effects of sertraline × cue for any of the locations (adjusted *p* > 0.1 in all cases)

The addition of a fish predator cue at Day 8 had a significant effect on both activity and location of the snails in all sertraline treatments. The percentage of active snails decreased from a mean of 48% in all treatments to 26% after addition of the cue (Fig. 1; *F*_1,19_ = 5.0, *p* = 0.038). However, there was no effect of sertraline on activity before or after the addition of chemical cue (*F*_3,16_ = 0.74, *p* > 0.1) and no interaction between the two (*F*_3,16_ = 0.17, *p* > 0.1). Predator cue also had a significant effect on the use of open habitats (Fig. 2a; *F*_1,19_ = 11, adjusted *p* = 0.011) for which the proportion of snails in open habitat decreased from 62% to 37%, but there was no significant effect of sertraline and no interaction effect (*F*_3,16_ = 1.4, *F*_3,16_ = 1.1, respectively, *p* > 0.1 in both cases). With regard to refuge-seeking behaviour, there was neither a significant effect of predator cue on proportion of snails at/above the surface (Fig. 2b; *F*_1,19_ = 4.9, adjusted *p* > 0.1), nor was there a significant effect of sertraline (*F*_3,16_ = 3.7, adjusted *p* > 0.1). There was also no interaction effect between the two stressors (*F*_3,16_ = 0.59, adjusted *p* > 0.1). The proportion of snails hiding under tiles increased approximately four-fold from 4% to 16% after addition of the cue (Fig. 2c; *F*_1,19_ = 5.39, adjusted *p* = 0.096), though this was not statistically significant at *α* = 0.05. There was no effect of sertraline (*F*_3,16_ = 0.49, adjusted *p* > 0.1) and no interaction between the two stressors (*F*_3,16_ = 0.93, adjusted *p* > 0.1).

### Boldness

**Fig. 3:**
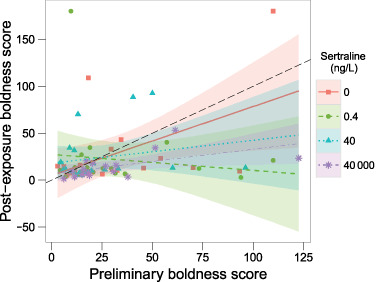
Data on the individual snails’ mean preliminary boldness scores (mean based upon 2 assays per snail before sertraline exposure; x-axis) vs. post-exposure boldness scores (assayed 1 time per snail on Day 7 of sertraline exposure; y-axis). Boldness scores indicate the amount of time (s) taken to re-emerge from the shell after mechanical stimulation (lower scores = bolder snails). Linear fits are plotted for each sertraline treatment (shading indicates the 95% CI) and the dashed, black line is the line of identity, indicating slope = 1 (i.e. no change in boldness score before and after sertraline exposure)

Snail boldness (time to re-emergence) showed high individual consistency between trials (Spearman’s *ρ* = 0.84, *p* < 0.001), although over time snails became bolder on average, indicated by the slopes of fit for each treatment being <1 (i.e. lower scores = less time to emerge from shell; Fig. 3). Sertraline exposure did not affect post-exposure boldness scores at Day 7 (*F*_3,16_ = 1.1, *p* > 0.1) nor the relative changes in boldness scores, i.e. pre- to post-exposure (*F*_3,16_ = 0.73, *p* > 0.1).

## Discussion

We did not detect consistent, dose-response related changes in activity pattern or habitat use of the freshwater snail *R. balthica* when exposed to the antidepressant sertraline over the exposure period. There was an indication of an interaction effect of sertraline and time on snail activity throughout Days 1 to 7 of exposure (*p* = 0.088; Supplementary material Fig. S1). Exposure to the two highest treatment concentrations may have reduced activity compared to the control between Days 3 and 5, though overall trends in effects of sertraline over time or across concentrations were not convincing. Hunger state in a different species of snail (*Lymnaea stagnalis*) of the same family as *R. balthica* has been shown to affect locomotion, in which recently-fed snails moved slower than hungry snails (Sidorov 2006). The lack of significant effects of time of day on snail activity in control animals (i.e. described in the Methods section under “Statistical Analyses”) makes it unlikely that feeding the snails every other day during the exposure period in our study affected the results of the activity measurements.

To our knowledge, there are no reports on organism-level effects of sertraline in gastropods. Sertraline generally has a high detection frequency in aquatic environments in Europe and North America (reviewed by Silva et al. 2012), with surface water concentrations measured up to 49 ng/L (Schultz and Furlong 2008) and wastewater effluents up to 120 ng/L (Nagarnaik et al. 2011). The highest sertraline concentration used in our study, 40 µg/L, was chosen because it is within the range shown to affect behaviour in fish (Hedgespeth et al. 2014; Valenti et al. 2012) and life history parameters in crustaceans (Henry et al. 2004; Lamichhane et al. 2014). Published studies on bioconcentration and bioaccumulation of pharmaceuticals in snails are scarce; the only report of a BAF for sertraline in snails found in the literature was 990 L/kg for snails from the family Planorbidae (Du et al. 2015). However, if *R. balthica* indeed displays a similar BAF for sertraline, we can assume that snails likely took up sertraline present in exposure media in our experiment.

For the more frequently studied SSRI fluoxetine, effect concentrations on reproductive parameters in gastropods have been reported in the low to high µg/L range (Nentwig 2007; Péry et al. 2008). The general lack of effects of sertraline on snails’ activity and other behaviours in this study is interesting considering that other SSRIs have been shown to induce changes in gastropod behaviour. For example, recent studies on locomotion after fluoxetine exposure showed effect concentrations of 20 µg/L for movement and burrowing behaviour in freshwater mussels (Hazelton et al. 2014), and of 40–345 µg/L for foot detachment and locomotion in marine snails (Fong et al. 2015; Fong and Molnar 2013). When considering human pharmacological data, one possible reason for this discrepancy might be that fluoxetine’s primary metabolite, norfluoxetine, is an equally effective SSRI whereas sertraline’s is inactive (van Harten 1993); additionally, though the two SSRIs have the same primary mechanism of action, fluoxetine’s binding is less selective for serotonin receptors (with more potential for dopamine and norepinephrine receptor interactions) compared with sertraline (Bymaster et al. 2002). The metabolism and receptor binding selectivities of various SSRIs in gastropods have not yet been assessed in depth and would help to elucidate apparent differences in sensitivity to different compounds. Interestingly, the reported toxic potency of sertraline to crustaceans has been higher than that of fluoxetine (Christensen et al. 2007; Henry et al. 2004); whether the opposite is true for gastropods still remains to be determined. Interspecies differences in sensitivity can vary by several orders of magnitude for SSRIs and are dependent upon the endpoints studied (e.g. reviewed by Silva et al. 2015); thus generalisations for both toxic levels and ranking across species should be made with caution.

We expected that sertraline would modulate the snails’ behavioural responses to predator cues (NCEs of the predators). Previous research has found that the enhanced memory formation and predator vigilance behaviour in *Lymnaea stagnalis* caused by predator cues via activation of the serotonergic system were blocked after serotonin receptor antagonists were injected (Il-Han et al. 2010). Regarding SSRIs specifically, a study on fluoxetine found that exposure to 3 µg/L eliminated hiding behaviour in tadpoles exposed to predator cues (Barry 2014), crayfish exposed to 2 and 500 µg/L fluoxetine demonstrated reduced locomotion (Tierney et al. 2016), and crabs exposed to 25 mg/L fluoxetine reduced the amount of time spent in a dark zone, indicating reduced anxiety-like behaviour (Hamilton et al. 2016). However, the observed effect of chemical cue from predatory fish on snail behaviour was independent of sertraline exposure in our study. When aquatic wastewater recipients contain molluscivorous fish, snails in these habitats are exposed to predators and chemicals like sertraline simultaneously. Our study organism *R. balthica* has been shown to reduce its activity and increase refuge use by either hiding under stones or climbing above the water surface as a response to chemical cues from predatory fish (Brönmark et al. 2012; Rundle and Brönmark 2001). In our study, fewer snails were present in the open after the addition of the chemical predator cue; snails responded to the cue by hiding underneath the tile and moving to or above the water’s surface (Fig. 2). Also, 20 min after the cue was added snails decreased their activity in all treatments by approximately 50% (Fig. 1). Sertraline did not significantly interfere with either refuge use (surface and under tile) or activity—i.e., at these concentrations and within the time-scale of the experiment, sertraline did not modify the NCEs induced by the predator cue.

As behavioural traits including aggression and social dominance are linked to serotonin levels in invertebrates (Kravitz and Huber 2003), we expected that SSRIs could also affect individual consistency in boldness. We predicted that sertraline exposure would alter boldness in *R. balthica* and consequently affect the snails’ predation risk by fish; however, we found no evidence for such effects in the current study. When studying the effects of biotic and abiotic stressors on prey behaviours, researchers typically focus on the “average” behaviours in a population even though there can be a large amount of variability among individuals in their behavioural responses to the stressor. This type of inter-individual variation in behavioural traits can be consistent over time and contexts within an individual, including the propensity to take risks (e.g. bold/shy continuum Bell et al. 2009; Wilson et al. 1994). This has also been observed in *R. balthica* for which shells are used as a refuge from predation and thus, relatively short emergence time from the shell could be considered a measure of boldness (Ahlgren et al. 2015). Further, such inter-individual differences in personality can play a key role in predator–prey interactions; for example, boldness can affect an individual snail’s probability of mortality by predation (Ahlgren J, et al., *unpublished data*). Similarly to Ahlgren et al. (2015), we found a high degree of consistency in time to emerge from the shell after mechanical stimulation meant to simulate potential predation risk, indicating that the assay is an effective means of measuring risk-taking behaviour in snails.

Regarding the mortality of the snails (24 ± 17% in the control treatment), our previous experience in working with *R. balthica* indicates that the level of mortality seen in this experiment is not unusually high. For example, a study published by Hallgren et al. (2012) demonstrated a mortality of *R. balthica* at 57 ± 15% in the control. The same study found a mortality ranging from 33–82% in various treatments of ethinylestradiol exposure with no significant difference between treatments. A separate study has also reported a mortality of 38% for *R. balthica* serving as experimental controls, i.e. snails uninfected with a parasite (Caron et al. 2007).

Though our study did not detect significant effects of sertraline resulting in impacts on snails’ predation risk, we suggest that such approaches including multiple stressors are nonetheless valuable to understanding ecological impacts of chemical contaminants. The uncertainties associated with the extrapolation of toxicity data from simplified, controlled laboratory studies used to predict risks in complex environments are profound. The sensitivity and susceptibility of organisms to chemicals can be affected by additional environmental or internal factors influencing bioavailability, toxicokinetics, or tolerance (Holmstrup et al. 2010). Chemical effects may therefore go unnoticed under optimal conditions and may only be expressed or observable when additional stress to the organism is present; for instance, studies on aquatic crustaceans have shown that exposure to multiple biotic and abiotic stressors can, as a result, lead to non-additive effects (Coors and De Meester 2008). To enable more realistic exposure and effect scenarios for organisms and ecosystems, researchers should continue to integrate multiple, natural and anthropogenic stressors in ecotoxicity testing (e.g. exposure to additional chemical contaminants, changes in abiotic factors like temperature or pH, or exposure to predators and predation risk).

## Conclusions

The general lack of effects of sertraline on snails’ activity and other behaviours in this study is interesting considering that other SSRIs have been shown to induce changes in gastropod locomotion (Fong et al. 2015). Additionally, sertraline exposure did not appear to impact NCEs induced by predator cues in *R. balthica*. Regarding the predator/prey system of this study, we suggest that predatory fish may be more sensitive to SSRIs than their snail prey. SSRIs have been shown to affect fish predator/prey behaviour at 0.01–4 µg/L concentrations (Dzieweczynski et al. 2016; Hedgespeth et al. 2014; Stanley et al. 2007; Valenti et al. 2012; Weinberger and Klaper 2014). Thus, given our findings and the current literature data on fish, we would predict a “trophic sensitivity mismatch” where predators may be affected at concentrations at which the anti-predator responses of gastropod prey remain unaffected. Additional experiments allowing for the predation of snails by fish would aid in determining whether the lack of sertraline’s impacts on NCEs also agrees with actual predation risk, as well as the subsequent influence thereof on freshwater communities.

## Electronic supplementary material

Supplementary Information

Supplementary Information
